# Pleomorphic dermal sarcoma: Clinicopathological features and outcomes from a 5‐year tertiary referral centre experience

**DOI:** 10.1002/cnr2.1583

**Published:** 2021-11-11

**Authors:** Ian T. Logan, Katherine M. Vroobel, Franel le Grange, Conal M. Perrett

**Affiliations:** ^1^ Department of Dermatology University College London Hospitals NHS Foundation Trust London UK; ^2^ Department of Histopathology University College London Hospitals NHS Foundation Trust London UK; ^3^ Department of Oncology University College London Hospitals NHS Foundation Trust London UK

**Keywords:** atypical, cutaneous sarcoma, fibroxanthoma, local recurrence, metastasis, pleomorphic dermal sarcoma, skin cancer

## Abstract

**Background:**

Pleomorphic dermal sarcoma (PDS) describes rare dermal‐based malignant tumours that are morphologically similar to atypical fibroxanthoma (AFX). PDS may be differentiated from AFX by the presence of one or more of the following histologic features: subcutaneous invasion, tumour necrosis, lymphovascular invasion (LVI), and/or perineural infiltration (PNI).

**Aims:**

To further define the clinicopathological features, surgical management, and outcomes of PDS primary tumours.

**Methods and Results:**

This study was a retrospective observational case series using a database search from 2012 to 2017. Inclusion criteria required all cases to meet the histopathologic criteria for PDS as confirmed by a specialist soft‐tissue histopathologist. A total of *n* = 17 cases were included with a median age of 78 years (range 66–85). All tumours were located on the head and neck, with 13/17 located on the scalp. Primary treatment was with wide local excision (WLE) in all cases. Median follow‐up was 48 months. Local recurrence occurred in 4/17 cases (24%) and distant metastasis in 2/17 cases (12%).

**Conclusion:**

PDS behaves more aggressively than atypical fibroxanthoma with which it shares a biologic continuum. The optimal surgical management approach is yet to be determined.

## 1 INTRODUCTION

Pleomorphic dermal sarcoma (PDS) describes rare dermal‐based malignant tumours that usually present on sun‐exposed sites of elderly patients and are morphologically similar to atypical fibroxanthoma (AFX). PDS may be differentiated from AFX by the presence of one or more of the following histologic features: subcutaneous invasion, tumour necrosis, lymphovascular invasion (LVI), and/or perineural infiltration (PNI).^1^, ^2^ The term PDS was proposed in 2012 by Prof. C. D. Fletcher to recognise that AFX‐like tumours with any of these morphological features are more likely to recur locally or metastasise than true AFX confined to the dermis.^1^

PDS was subsequently recognised as a distinct diagnostic entity in the 2013 revision of the World Health Organisation Classification of Soft Tissue Tumours in which the term ‘malignant fibrous histiocytoma’ was discarded.^2^, ^3^ The terms ‘superficial malignant fibrous histiocytoma’ and ‘undifferentiated pleomorphic sarcoma of skin’ have previously been used to describe tumours morphologically similar to what may now be reported as PDS.^4^, ^5^, ^6^, ^7^, ^8^, ^9^

Local recurrence in case series of AFX occurs in approximately 10% of patients, with regional or distant metastasis occurring in <4% of cases.^4^, ^10^, ^11^ Case series of PDS have reported local recurrence rates from 7‐35% and distant metastases in 2–20% of cases.^4^, ^12^, ^13^, ^14^, ^15^, ^16^ To describe further the clinicopathological features and surgical management of this recently reclassified entity, we completed a study of PDS cases managed at our institution over a 5‐year period.

## 2 METHODS

This study was a retrospective observational case series conducted at the Departments of Dermatology and Oncology, University College London Hospitals NHS Trust, which is part of the London Sarcoma tertiary referral service. The study protocol conformed to the ethical guidelines of the 1975 Declaration of Helsinki and study authorisation was granted by the Healthcare Research Authority following research ethics committee approval. Cases were considered for inclusion following a search of our pathology and sarcoma multi‐disciplinary team (MDT) records for diagnoses coded as; AFX or PDS. The search was restricted to the years January 2012–January 2017 to allow a minimum of 2 years clinical follow‐up. The histology report content of identified cases was reviewed by the study investigators.

Inclusion criteria for the study required the primary tumour to have been confirmed by a specialist soft tissue histopathologist to meet the histologic criteria for PDS demonstrating: a dermal‐based tumour with histologic and immunohistochemical features of AFX in addition to evidence of diffuse invasion of subcutaneous adipose tissue and/or tumour necrosis, PNI, LVI. Lack of staining by immunohistochemistry for all cytokeratin and melanocytic markers tested as well as desmin and CD34 was required, with evidence of at least one pancytokeratin and melanocytic marker tested. Routine haematoxylin and eosin (H + E) stained sections had previously been reported by two histopathologists for concordance. Immunohistochemical studies had been performed following standard protocols at both the referring institutions and the London Sarcoma Service (Royal National Orthopaedic Hospital) for correlation. Patients living with PDS with less than 2 years follow‐up from excision of the primary tumour were excluded. Any patients dying from metastatic PDS within 2 years follow‐up were included.

Clinical and histopathological data were extracted from the case records, imaging systems, pathological record database, and by communication with the referring institution or general practice by the study investigators. Primary outcomes were the development of local recurrence (LR) defined as recurrence within 2 cm of the surgical site, and metastatic disease. Metastatic disease was categorised as; in‐transit metastasis (ITM) when present >2 cm away from the primary tumour site but before reaching the nearest lymph node, regional, or distant. Histologically, tumour margins were categorised as clear if the tumour was ≥1.0 mm clear of the margin, or narrow if <1.0 mm.

## 3 RESULTS

The search identified 20 cases of PDS from the sarcoma MDT database from the period January 2012–December 2016. One case of which had been reclassified as AFX on secondary review. Two further cases were excluded as the PDS diagnosis had been made on recurrent tumours following excision of an AFX primary. The pathology database search identified additional cases of AFX (*n* = 6) none of which met the histopathologic criteria for reclassification as PDS. Therefore 17 patients met the inclusion criteria (*n* = 17), nearly all of which were elderly males with a male to female ratio of 16:1. The median age was 78 years (range 66–85 years). The Fitzpatrick skin phototype was I‐II in all patients except one patient of phototype III. A summary of the clinicopathological features is detailed in Table 1.

**TABLE 1:** Summary of clinicopathological features

Case number	Age	Site	Size	Duration/months	Depth	Tumour necrosis	LVI	PNI	Stage at resection
1	82 m	Eyebrow	15 mm	unclear	Skeletal muscle	_	_	unreported	pT1b NxMxG3
2	67 m	Forehead	15 mm	3	Deep subcutis	_	_	unreported	pT1aNxMxG3
3	80 m	Scalp	14 mm	unclear	Deep subcutis	+	_	+	pT1aNxMxG3
4	85 m	Scalp	unclear	unclear	Deep subcutis	unreported	unreported	unreported	pT1aNxMxG3
5	66 m	Scalp	13 mm	8	Deep subcutis	_	unreported	unreported	pT1aNxMxG3
6	85 m	Eyebrow	45 mm	6	Deep subcutis	+	+	unreported	pT2aNxMxG3
7	73 m	Scalp	20 mm	3	Deep subcutis	_	+	+	pT1aNxMxG3
8	68 m	Scalp	9 mm	4	Deep subcutis	_	_	unreported	pT1aNxMxG3
9	81 m	Scalp	15 mm	2	Deep subcutis	_	_	+	pT1aNxMxG3
10	78 m	Scalp	25 mm	5	Deep subcutis	unreported	_	_	pT1aNxMxG3
11	80 m	Scalp	24 mm	unclear	Deep dermis	_	_	+	pT1aNxMxG3
12	78 m	Scalp	47 mm	unclear	Deep subcutis	_	_	_	pT2aNxMxG3
13	80 m	Scalp	30 mm	unclear	Deep subcutis	unreported	+	unreported	pT1aNxMxG3
14	69 m	Scalp	15 mm	1	Deep subcutis	+	_	+	pT1aNxMxG3
15	70 m	Scalp	20 mm	8	Fascia	unreported	+	unreported	pT1bNxMxG3
16	81 f	Forehead	18 mm	unclear	Deep subcutis	unreported	_	_	pT1aNxMxG3
17	78 m	Scalp	27 mm	2	Deep subcutis	unreported	unreported	unreported	pT1aNxMxG3
Median	78	n/a	19 mm	3.5	n/a	n/a	n/a	n/a	n/a

*Note*: + confirmed present – confirmed absent.

Abbreviations: AWOD, alive without disease; LR, local recurrence; DWOD, died without disease; DFD, died from disease; m, male; f, female; LVI, lymphovascular invasion; PNI, perineural infiltration; TNM, tumour node metastasis stage; G, tumour grade.

**FIGURE 1:**
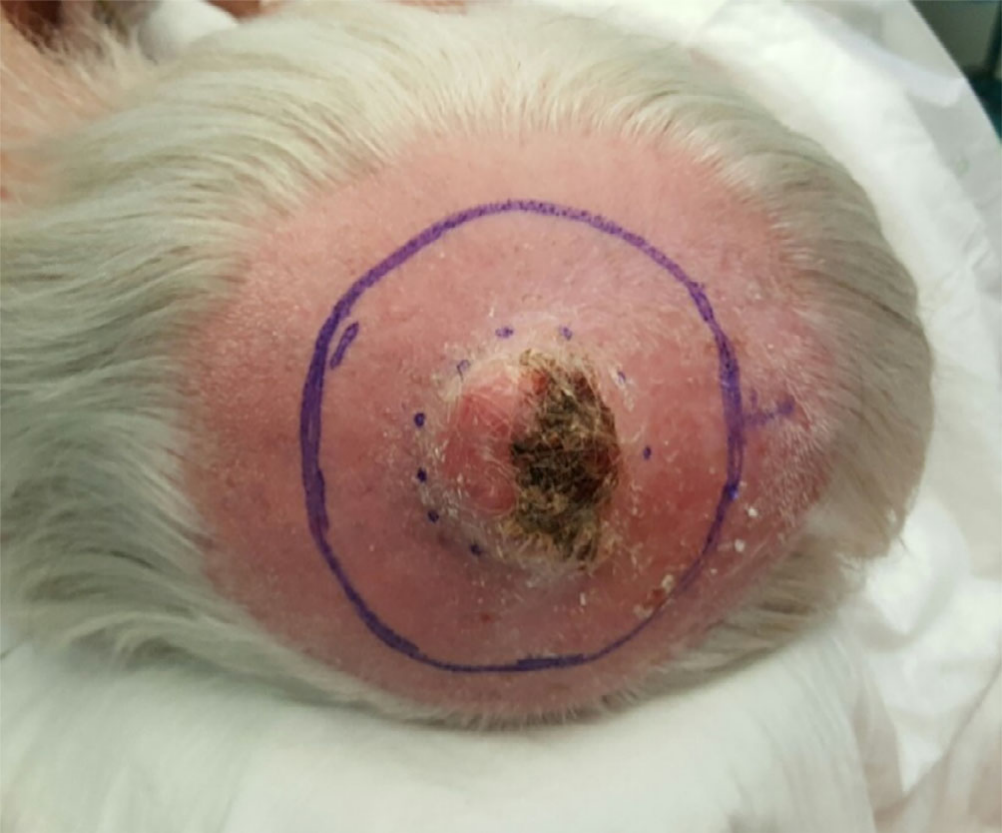
Clinical image of a PDS exophytic nodule of the scalp prior to WLE. PDS, pleomorphic dermal sarcoma; WLE, wide local excision

The scalp was the most frequent tumour location (*n* = 13) followed by the forehead (*n* = 2) and eyebrow (*n* = 2). The median tumour size was 19 mm (range 9–47 mm). Tumours presented as nodules (Figure 1) in the majority. Tumour surface characteristics were ulcerated (*n* = 12) non‐ulcerated (*n* = 2) and unknown (*n* = 3). The median duration of nodule growth was 3.5 months (range 1–8 months). The primary tumour clinical diagnosis was most commonly squamous cell carcinoma (*n* = 6), followed by basal cell carcinoma (*n* = 3), unknown (*n* = 5), or a differential diagnosis including two or more of; BCC, SCC, AFX, amelanotic melanoma, or Merkel cell carcinoma (*n* = 3). No patients had clinically detectable local or regional lymphadenopathy at presentation. Prior history, or additional diagnosis at presentation, of ultraviolet (UV)‐associated pre‐malignant lesions (actinic keratosis, Bowen's disease) or non‐melanoma skin cancer was found in 14/17 patients (82%). No patients had visceral malignancy, other than three patients receiving follow‐up for prostate cancer treatment. One patient (case 17) had previously received azathioprine for ulcerative colitis, and one patient (case 2) had received prednisolone courses for interstitial lung disease.

### 3.1 Surgical management

For *n* = 17, patients were initially investigated either by diagnostic punch biopsy (*n* = 4), incisional biopsy (*n* = 5) or wide local excision (*n* = 8). All patients received primary treatment with a WLE, surgical margins ranging from 4 to 10 mm (*n* = 11) but not clearly documented in six patients.

The surgical management and pathological tumour margins are detailed in Table 2. Following primary surgical excision, the peripheral histologic margins were ≥1 mm clear in 16/17 patients, and the deep margin was clear by a narrow margin in 4/17 patients and involved in 1/17 patients. A second WLE was performed in patients with involved or narrow margins (*n* = 4), or declined on patient preference (*n* = 1), or not achievable due to local recurrence at 1 month (case 7). Therefore, 15/17 patients achieved complete margin clearance of the primary tumour.

**TABLE 2:** Surgical management, histopathological margins and outcomes

Case number	Primary treatment	Peripheral histopathologic margin	Deep histopathologic margin	Secondary treatment of primary tumour	Primary tumour resection status (total surgical margin)	Local recurrence	Local recurrence treatment	Outcome
1	WLE 4 mm	1.2 mm	Involved	WLE 5 mm	R0 (9 mm)	N	‐	AWOD
2	WLE 4 mm	2 mm	1.2 mm	n/a	R0 (4 mm)	Y – 7 mo	WLE ‐ nfr	Lung metastases 7mo. DFD 9mo.
3	WLE*	3.7 mm	0.6 mm	WLE 10 mm	R0 (>10 mm)	N	‐	AWOD
4	WLE 10 mm	No tumour	No tumour	n/a	R0 (10 mm)	N	‐	AWOD
5	WLE 10 mm	5 mm	0.5 mm	WLE 20 mm	R0 (30 mm)	N	‐	AWOD
6	WLE*	6 mm	2 mm	n/a	R0 (unclear)	N	‐	AWOD
7	WLE 5 mm	7 mm	0.8 mm	n/a	R1 (5 mm)	Y‐ 1 mo	WLE − +ve deep margin. Adjuvant RTx.	AWOD 58mo
8	WLE*	5.8 mm	1.8 mm	n/a	R0 (unclear)	N	‐	AWOD
9	WLE 10 mm	13 mm	1.5 mm	n/a	R0 (10 mm)	Y – 1 mo	WLE ‐ nfr	AWOD 59mo
10	WLE*	4 mm	7.5 mm	n/a	R0 (unclear)	N	‐	DWOD
11	WLE 6 mm	3 mm	1 mm	n/a	R0 (6 mm)	N	‐	DWOD
12	WLE 10 mm	10 mm	1.5 mm	n/a	R0 (10 mm)	Y – 3mo + 4mo	WLE + ve deep margin. Palliative brachytherapy.	ITM + Lung metastases 12 months. DFD 43 months.
13	WLE 10 mm	6 mm	4 mm	n/a	R0 (10 mm)	N	‐	AWOD
14	WLE*	Involved	0.2 mm	WLE 10 mm	R0 (>10 mm)	N	‐	AWOD
15	WLE 6 mm	7 mm	0.5 mm	n/a	R1 (6 mm)	N	‐	AWOD
16	WLE 6 mm	7 mm	2 mm	n/a	R0 (6 mm)	N	‐	AWOD
17	WLE*	5 mm	1 mm	n/a	R0 (unclear)	N	‐	AWOD

*Note*: WLE, wide local excision (surgical margin as specified); WLE*, wide local excision mm margin not documented; LR, local recurrence; Y, yes; N, no; AWOD, alive without disease; DWOD, died without disease; DFD, died from disease; ITM, in‐transit metastases; nfr, no further recurrence; n/a, not applicable; R0 = pathologic margins ≥1.0 mm R1 = pathologic margins <1.0 mm.

**FIGURE 2:**
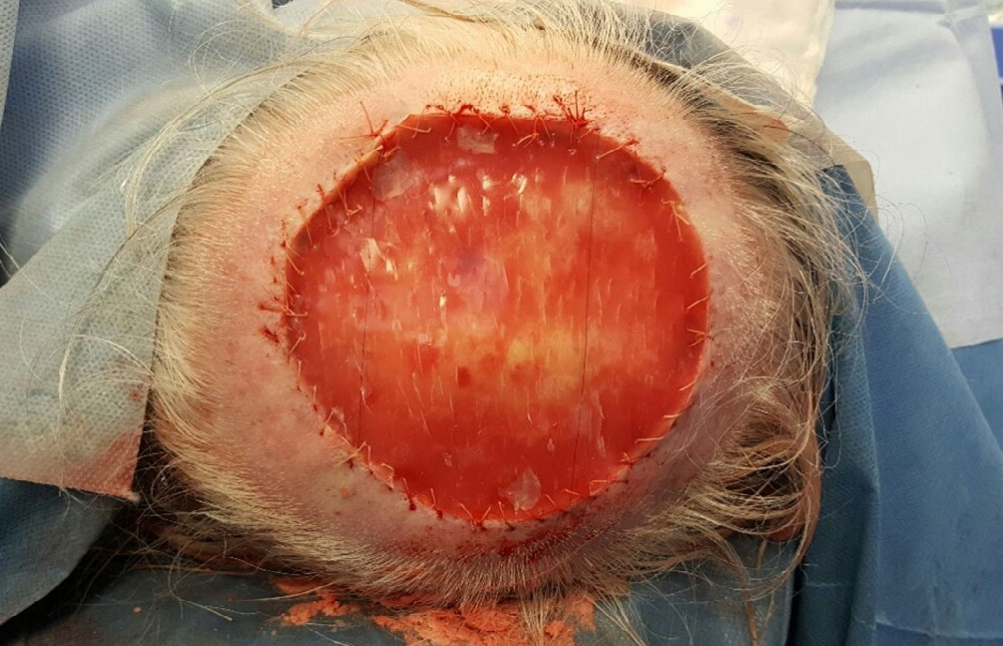
Full‐thickness scalp defect following wide local excision repaired with an acellular dermal matrix

Full thickness scalp surgical defects ranging from 3 to 10 cm diameter in this series were repaired by secondary intention healing (*n* = 3), split‐thickness skin graft (SSG; *n* = 3), local flap (*n* = 1), full‐thickness skin graft (*n* = 8), or a combination of acellular dermal matrix application (Figure 2) followed by negative pressure dressing (VAC) and subsequent SSG application (*n* = 2).

### 3.2 Histopathological features

**FIGURE 3:**
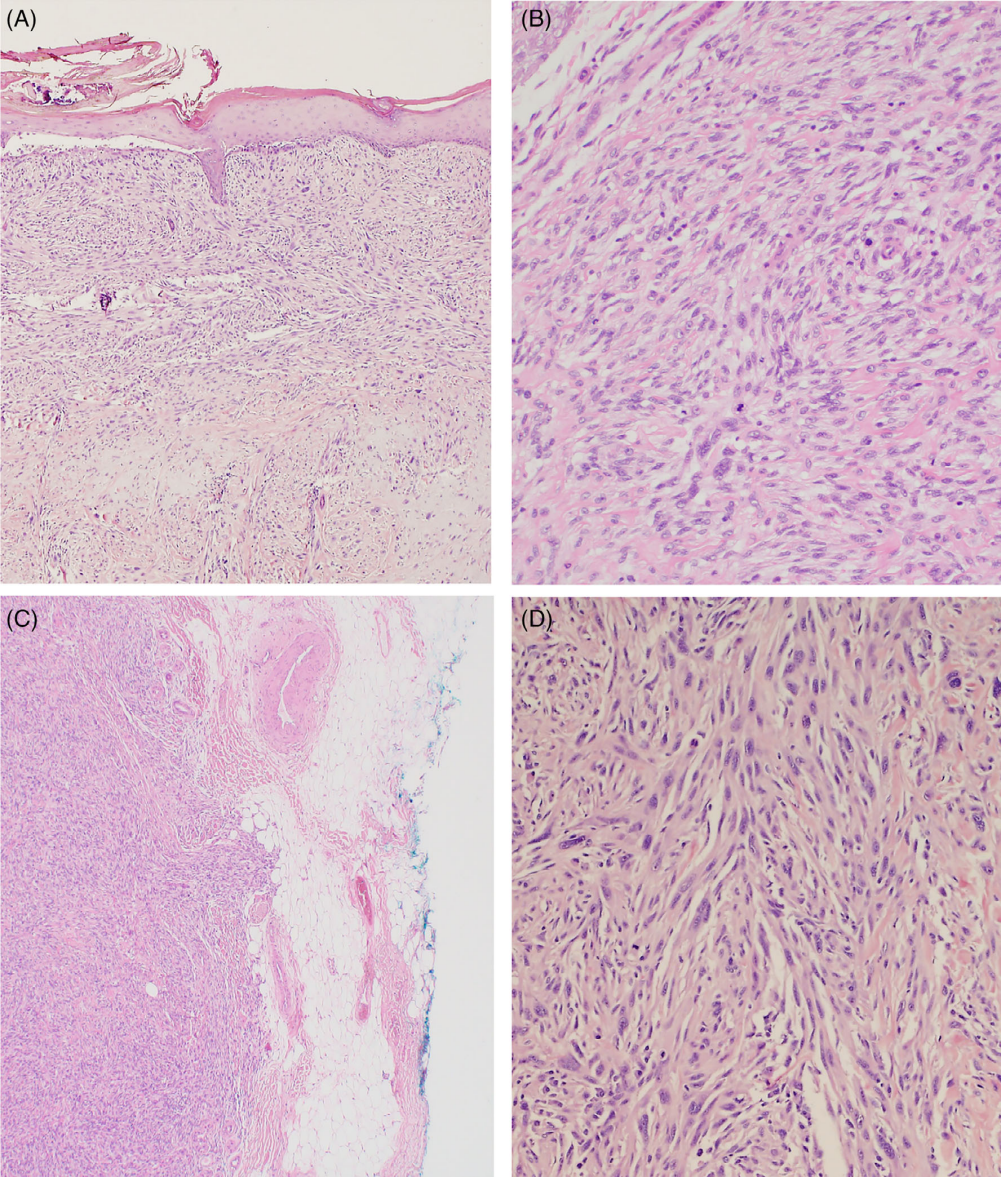
Haematoxylin and eosin (H + E) stains of pleomorphic dermal sarcoma. (A) Pleomorphic dermal sarcoma (PDS) a dermal spindle cell tumour extending to the overlying epidermis (original magnification ×40). (B) Pleomorphic epithelioid spindle cells typical of PDS (original magnification ×200). (C) PDS demonstrating a pushing border into the subcutis (original magnification ×40). (D) The same tumour as in 3a. PDS demonstrating the typical fascicular growth pattern (original magnification ×200) [Correction added on 7 November 2022, after first online publication: The Figure 3 citation and legend have been corrected in this version.]

All tumours were atypical spindle cell tumours originating within the dermis usually with extensive dermal solar elastosis. The predominant morphological pattern was of markedly pleomorphic spindle cells (Figure 3) arranged in fascicles (Figure 3d, photomicrograph from the same slide as 3a). Depth of invasion was as follows: dermis only = 1, subcutaneous adipose = 14, fascia = 1 and muscle = 1. The type of invasion was described as infiltrative = 9, predominantly pushing = 3, nodular and honeycomb = 1, and unspecified = 4.

**FIGURE 4:**
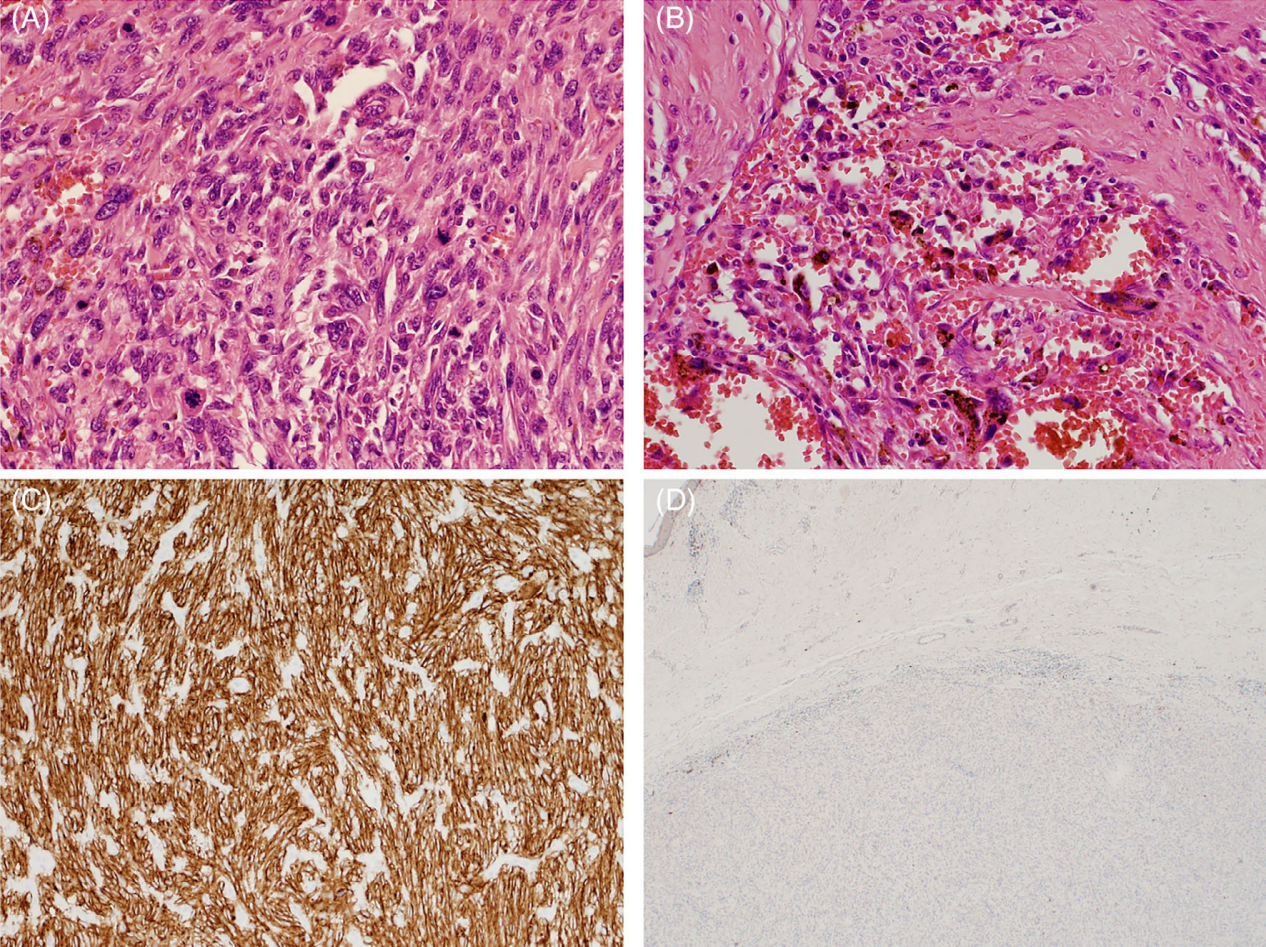
Histopathological images of PDS demonstrating associated features and IHC staining. (A) Atypical mitotic figures (H + E original magnification ×200). (B) Intratumoral haemorrhage and haemosiderin (H + E original magnification ×200). (C) IHC stain demonstrating diffuse positive staining to CD10 (original magnification ×200). (D) IHC stain demonstrating negative staining to S100 (original magnification ×40). IHC, immunohistochemical; PDS, pleomorphic dermal sarcoma

Brisk or very marked mitotic activity was reported in 16/17 cases, typically with a mitotic count of >20/10 high power field (HPF). Atypical mitoses were frequently noted (Figure 4A), and less commonly intratumoral haemorrhage (Figure 4B). Tumour necrosis was identified in 3/17 cases (18%), not identified in 8/17 cases, and not reported on in the remainder. LVI was identified in 4/19 cases (22%), not identified in 10/17 cases, and not reported on in the remainder. PNI was identified in 4/17 cases (24%), entrapment without infiltration in 2/17 cases, not identified in 2/17 cases, and not reported on in the remainder.

A summary of the immunohistochemistry staining profile of the tumours is detailed in Table 3. Positive staining to CD10 was found uniformly (Figure 4C), and negative staining to melanocytic markers in all tumours (Figure 4D).

**TABLE 3:** Summary of the immunohistochemistry staining profiles

Case number	MNF116	AE1/3	CK5/6	p63	S100	Melan A	HMB‐45	SMA	Desmin	CD10	CD34	CD31	CD68
1	−	n/a	n/a	n/a	−	−	n/a	−	−	+	−	n/a	n/a
2	−	−	−	−	−	−	−	−	−	n/a	−	−	n/a
3	−	−	−	−	−	−	−	−	−	+	−	−	+
4	−	−	n/a	−	−	−	−	−	−	+	−	n/a	n/a
5	−	−	−	−	−	−	n/a	−	−	+	−	−	+
6	−	n/a	−	−	−	−	n/a	+	n/a	+	−	−	n/a
7	−	−	−	−	−	−	n/a	+	−	+	−	+	+
8	−	−	n/a	−	−	−	n/a	−	−	+	−	−	n/a
9	−	−	−	−	−	−	−	+	−	+	n/a	−	n/a
10	−	−	−	−	−	−	−	+	−	n/a	−	−	+
11	−	−	n/a	−	−	−	−	+	−	+	n/a	n/a	+
12	−	−	n/a	n/a	−	−	−	−	−	+	n/a	n/a	n/a
13	−	−	n/a	−	−	n/a	−	+	−	+	n/a	−	n/a
14	−	n/a	−	−	−	−	−	−	−	+	−	n/a	n/a
15	−	−	n/a	−	−	n/a	−	−	−	n/a	n/a	−	+
16	−	−	n/a	−	−	−	n/a	+	−	+	−	n/a	n/a
17	−	−	n/a	−	−	−	n/a	+	−	+	−	n/a	n/a

*Note*: −, negative staining; +, positive staining; n/a, not performed.

### 3.3 Clinical follow‐up

Patients were all followed up initially by the London Sarcoma Service and then subsequently at the referring institution in select cases for patient choice. The median follow‐up was 48 months (range 9–88 months). Patients were reviewed clinically for signs of locoregional recurrence, or symptoms of metastatic disease at 3‐month intervals for the first 2 years, and then at 6‐monthly intervals until 5 years, and then annually up to 10 years as per local protocol. A staging computed tomography (CT) chest without contrast was performed following diagnosis, and subsequently screening plain film chest X‐rays were performed during all clinical follow‐up visits.

Local recurrence occurred in 4/17 cases (24%), and distant metastases in 2/17 patients (12%). Local recurrences occurred between 1 and 7 months after initial surgery (median 2 months). For *n* = 4, the primary tumour had been removed with clear histological margins in three patients, with one patient (case 7) having a narrow deep margin at 0.8 mm. Recurrences were described as papules or nodules ranging from 5 to 30 mm in diameter. All were managed with WLE taken down to bone (10 mm peripheral margin = 1, 20 mm peripheral margin = 3). Clearance was achieved in two patients (case 2 and 9) with no further local recurrence. Case 2 developed biopsy proven metastatic disease at 7 months and has been reported previously.^17^ Case 7 had deep margin involvement of the local recurrence WLE and was treated with adjuvant radiotherapy 50Gy in 30# over 12 weeks without further recurrence. Case 12 developed local recurrence and multiple nodules of in‐transit metastasis. Wide local excision (specimen 110 x 50 mm) was unable to clear the tumoral deposits at 3 months with deep margin involvement. Post‐operative brachytherapy was used in the form of 36Gy in 6# twice a week. Computed Tomography (CT) chest scan at 5 months identified lung nodules, but no avid disease was demonstrated on positron emission tomography/computed tomography scan (PET CT) scan. Repeat PET CT scan at 12 months identified multiple lung metastases. The patient declined palliative chemotherapy and was treated with palliative dexamethasone and subsequently died from disease at 43 months.

## 4 DISCUSSION

Pleomorphic neoplasms typically have a high‐grade malignant potential but this does not readily apply to primary cutaneous tumours.^18^ This paradigm is demonstrated by AFX which is a distinctive cutaneous mesenchymal neoplasm characterised by morphologic features of malignancy but benign or extremely low‐grade malignant clinical behaviour.^6^, ^18^ PDS and AFX exist along a biologic continuum and are dichotomised by histologic features which have been demonstrated to be indicators of more aggressive biologic behaviour.^1^, ^2^, ^12^, ^13^ Both PDS and AFX predominantly affect the sun‐damaged head and neck of elderly males, and this was the similar finding for PDS in our series.^5^, ^12^, ^13^, ^16^, ^18^, ^19^, ^20^

Histologically, PDS is a dermal‐based tumour with pushing and infiltrative growth, characterised by a sheet‐like and fascicular growth of pleomorphic histiocyte‐like, spindled, and multinucleated tumour cells with brisk and atypical mitotic activity.^18^ There is no specific immunohistochemical (IHC) stain for PDS or AFX which remain diagnoses of exclusion.^2^ Typically IHC findings are positive for vimentin, CD10, CD68, and actin, and should be negative for pancytokeratins, CD34, melanoma markers melanA, S100 protein, and HMB45 as demonstrated in Table 3.^16^, ^21^ CD10 negativity should suggest the possibility of an alternative diagnosis.^12^, ^13^, ^22^ The histopathological differential diagnosis includes but is not limited to: desmoplastic melanoma, spindle cell squamous cell carcinoma, angiosarcoma, and leiomyosarcoma. A specialist soft tissue pathologist opinion is recommended.

Both AFX and PDS have been shown to frequently harbour mutations in FAT1, NOTCH1/2, CDKN2A, TP53, and the TERT promoter with similar mutation profiles between the tumours.^23^ Genetic studies have demonstrated the presence of UV‐signature mutations P53, HRAS, and KRAS in PDS tissue.^21^, ^24^ In the absence of differentiating genetic features, distinguishing AFX from PDS is at present therefore still best performed histologically.^23^

The depth or degree of subcutaneous invasion required to differentiate AFX from PDS is subject to inter‐observer variation, and complicated further by the thin subcutis present on the head and neck. Miller et al and Tardio et al both used ‘deep subcutaneous invasion’ as inclusion criteria for their case series but the requirement for ‘deep invasion’ is absent from the published WHO classifications.^2^, ^12^, ^13^, ^25^ The relevance of diagnostic dichotomisation is primarily the recognition of the higher likelihood of local recurrence, distant metastasis and tumour related mortality with which PDS is associated in comparison to AFX.^1^, ^6^, ^12^, ^13^, ^17^ The common pattern for progressive disease is multiple cutaneous metastases and lung parenchymal disease which is typical of haematogenous dissemination of sarcoma.^12^, ^13^

We present to our knowledge the sixth retrospective case series on PDS.^12^, ^13^, ^14^, ^15^, ^16^ Our study is the first to have patients identified for inclusion by using a departmental sarcoma MDT database, a specialist sarcoma histopathologist assessment of all cases, and with complete follow‐up. Previous studies have used a reclassification of historic cases of AFX as sourcing methodology, including from a period prior to PDS definition (2003–2012). Heterogeneity in the proportion of AFX tumours reclassified is likely to influence reported outcomes making comparisons difficult.^12^, ^13^, ^15^, ^16^ We also searched our hospital pathology database for cases of AFX that may be eligible for reclassification as PDS, but this did not generate any eligible cases.

Our report of the metastatic disease development in 2/17 patients (12%) is reflective of the malignant potential of this dermal neoplasm, falling midway between 2 and 19% of previous studies.^12^, ^13^, ^14^, ^15^, ^16^ Metastasis to the lung was noted in both patients, which is established as the most common site for distant metastasis in soft‐tissue sarcomas (STS).^13^, ^17^, ^26^ The main limitation of our study is the small sample size, and lower limit of clinical follow‐up period of 31 months for surviving patients.

Management and histological assessment of cases in specialist centres for sarcomas is recommended.^26^, ^27^ The increased absolute risk of distant metastatic disease in patients with local recurrence, and the increased risk of local recurrence in patients with narrowly or incompletely excised primary tumours suggests that aggressive and expedient surgical management to achieve tumour clearance and local control should be the primary treatment objective.^4^, ^12^, ^13^ We can make no statistically significant recommendations about the optimal surgical approach due to the sample size and the absence of a comparison group treated with Mohs micrographic surgery. No tumours were noted to recur in *n* = 4 patients that had a second WLE procedure which was offered due to histologic involved or narrow (<1 mm) deep margins. Achieving increased tumour clearance from the deep margin is a major challenge for managing PDS considering the typical anatomical locations. Wide Local Excision with a 20 mm margin has been recently reported to be associated with a superior progression free survival in a multi‐centre cohort review by Persa et al.^28^ The efficacy of adjuvant treatments such as radiotherapy requires further definition. The role of sentinel‐lymph node biopsy has also not been established.^29^ The optimal adjuvant and metastatic disease management is also not yet clear, but complete remission following anti‐PD‐1 immune checkpoint inhibitor Pembrolizumab treatment has been recently reported.^30^

PDSs continue to represent a diagnostic and clinical management challenge. Uniform adoption and application of soft tissue sarcoma classifications and minimum reporting datasets will promote homogeneity in clinicopathological analysis. Further research to compare the long‐term outcomes of WLE versus Mohs micrographic surgery is required. Aggressive surgical management to prevent local recurrence, clinical surveillance, and multidisciplinary management following diagnosis of PDS remains the mainstay of treatment.

## CONFLICT OF INTEREST

The authors declare no conflict of interest.

## AUTHOR CONTRIBUTIONS

*Data Curation, Formal Analysis, Investigation, Methodology, Project Administration, Resources, Software, Validation, Visualization, Writing—Original Draft, Writing – Review and Editing*, I.L.; *Data Curation, Formal Analysis, Investigation, Methodology, Resources, Software, Validation, Writing—Original Draft, Writing—Review and Editing*, K.V.; *Formal Analysis, Investigation, Methodology, Validation, Writing—Review and Editing*, F.G.; *Conceptualization, Data Curation, Formal Analysis, Investigation, Methodology, Project Administration, Supervision, Validation, Writing—Review and Editing*, C.P.

## ETHICAL STATEMENT

The study protocol conformed to the ethical guidelines of the 1975 Declaration of Helsinki. Study authorisation was granted by the Healthcare Research Authority following research ethics committee approval. Through anonymisation of patient identifiable data the requirement for individual patient consent was waived.

## Data Availability

The data that support the findings of this study are available from the corresponding author upon reasonable request.
